# Cardio-audio synchronization drives neural surprise response

**DOI:** 10.1038/s41598-017-13861-8

**Published:** 2017-11-01

**Authors:** Christian Pfeiffer, Marzia De Lucia

**Affiliations:** Laboratoire de Recherche en Neuroimagerie (LREN), University Hospital (CHUV) and University of Lausanne (UNIL), Lausanne, Switzerland

## Abstract

Successful prediction of future events depends on the brain’s capacity to extract temporal regularities from sensory inputs. Neuroimaging studies mainly investigated regularity processing for exteroceptive sensory inputs (i.e. from outside the body). Here we investigated whether interoceptive signals (i.e. from inside the body) can mediate auditory regularity processing. Human participants passively listened to sound sequences presented in synchrony or asynchrony to their heartbeat while concomitant electroencephalography was recorded. We hypothesized that the cardio-audio synchronicity would induce a brain expectation of future sounds. Electrical neuroimaging analysis revealed a surprise response at 158–270 ms upon omission of the expected sounds in the synchronous condition only. Control analyses ruled out that this effect was trivially based on expectation from the auditory temporal structure or on differences in heartbeat physiological signals. Implicit neural monitoring of temporal regularities across interoceptive and exteroceptive signals drives prediction of future events in auditory sequences.

## Introduction

When exposed to sensory inputs within sequence regularities, the brain generates an expectation that the same rules will apply to future sensory events. This expectation is revealed by measuring the brain response to regularity violation because of unexpected stimuli or sequence interruptions. A paradigmatic example is the electroencephalographic (EEG) response to unexpected versus expected stimuli that manifests as a negative modulation at fronto-central electrodes (i.e. mismatch negativity, MMN; see^1,2^ for reviews), a fundamental marker of sensory-memory formation that can be elicited pre-attentively and even in unconscious individuals^3–5^. While the MMN literature has largely focused on violation of stimulus type^1,6–12^, recent studies used omission of stimuli in order to directly measure the consequences of predictions generated by internal expectation in the absence of concurrent sensory input^13–17^. One main interest in studying neural responses to omission is they provide a direct demonstration of ‘predictive coding’^1,9,18,19^, according to which a hierarchy of generative brain models actively infers the most likely sensory input based on previous sensory experiences^20^. According to this theory, a functional hierarchy of neurons compares top-down predictions to bottom-up information in order to estimate prediction error.

The majority of violation detection studies investigated the omission response using regular sequences and fixed interstimulus intervals (see^1^ for a review). Moreover recent studies highlight the importance of temporal prediction for sensory-memory trace formation induced by regular sequences^21–24^. At present, violation detection and its dependence on stimulus predictability over time was mainly studied by establishing regularities within unimodal auditory (see^2^ for a review), somatosensory^10,25–27^ or visual stimulus sequences^9,19,28^, and more recently across different sensory modalities^29–31^. However, no study has tested whether temporal regularity between internal bodily processes and external stimuli could induce stimulus predictability, thus violation detection upon stimulus omission.

Recent theories based on predictive coding stress a major contribution of interoceptive signals (from inside the body, i.e. visceral, respiratory, cardiac) to time and body perception^32,33^. These proposals received empirical support by behavioral and neuroimaging studies in humans in which temporal synchronization of exteroceptive (from outside of the body, i.e. acoustic, visual) and heartbeat or respiratory signals modulated stimulus processing and bodily awareness^34–38^. Based on these results, we hypothesized that synchronization of interoceptive-exteroceptive signals can also impact the prediction of upcoming sensory stimuli. Specifically, the aim of this study was to identify whether omission responses would be observed in sound sequences temporally locked to heartbeats but with variable interstimulus interval.

We tested this hypothesis by manipulating the temporal synchronization between sound sequences and the participant’s heartbeat, using either a fixed (Synchronous condition) or a random (Asynchronous condition) temporal interval. This paradigm allowed studying violation detection in irregular unimodal sequences that were matched for auditory temporal structure and cardiac parameters across experimental conditions. Thus, in the Synchronous condition when predictability of sound onset locked to heartbeats was high, the omission of predicted sounds should induce prediction error manifesting in a violation detection, or omission response^13,15,16^. By contrast, in the Asynchronous condition where sound predictability based on heartbeats was low, a much weaker or even absent omission response is expected. We recorded EEG from human participants to analyze the neural responses during expected but omitted sounds. We hypothesized to observe a stronger violation detection response to sound omission during temporally synchronous than asynchronous cardio-audio stimulation as a consequence of an implicit cardio-audio temporal rule established in the Synchronous condition. Collectively, our paradigm aims at providing the first demonstration of violation detection induced by cardio-audio synchronization in non-isochronous auditory sequences based on individually adjusted heartbeat rhythms.

## Results

### Omission Response Analysis

**Figure 1:**
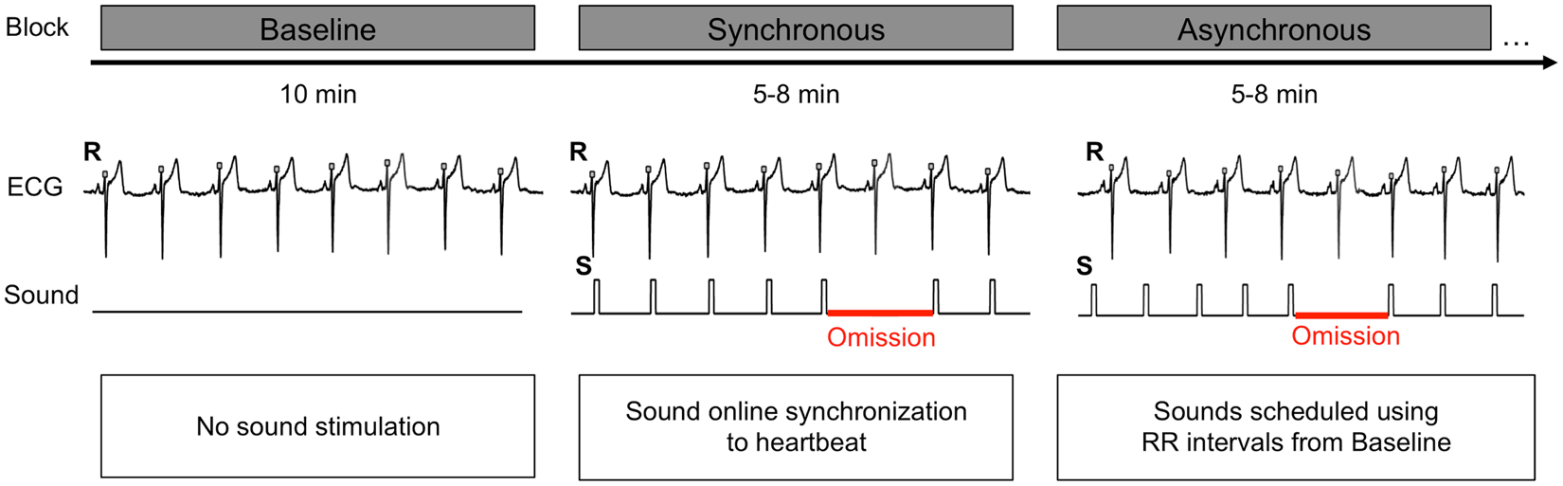
Experimental design and procedure. Exemplar experimental block order for the initial three out of a total of six blocks are shown in the top row. Electrocardiography (ECG) recordings superimposed with detected R peaks (i.e. R) are shown alongside with sound stimulation periods (S). Sound omission periods are highlighted in red color. Description of the sound stimulation methods for each experimental block is indicated at the bottom.

**Figure 2:**
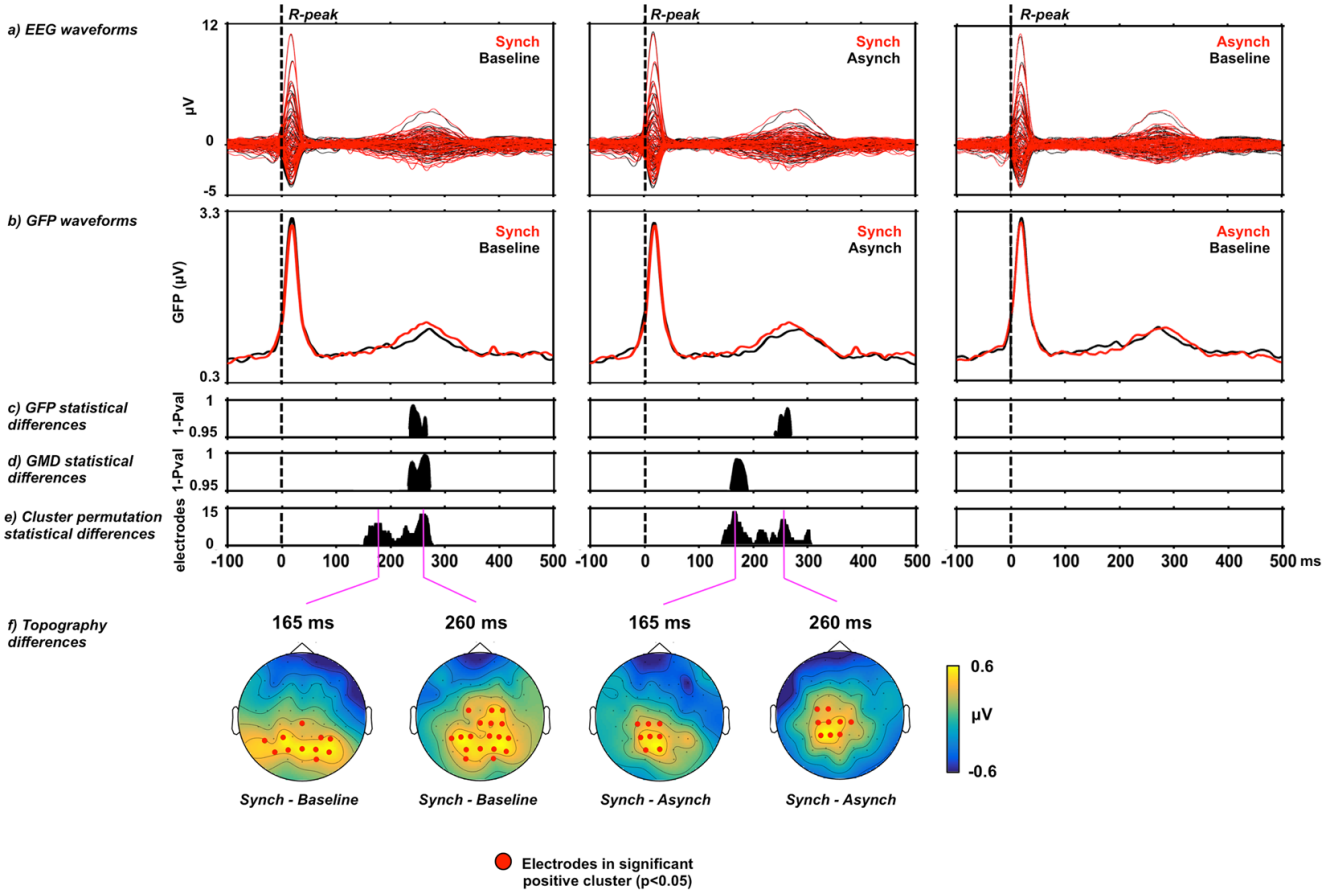
Electrical neuroimaging results for Heartbeat-Evoked Potentials (HEP) during sound omission. (**a**) Group-average EEG waveforms (N = 16) showing HEPs for the comparison of Synchronous (in red) versus Baseline trials (in black; left column), for Synchronous (in red) versus Asynchronous trials (in black; center column), and for Asynchronous (in red) versus Baseline (in black; right column). (**b**) Group-average Global Field Power waveforms (GFP) (**c**) GFP statistical differences (p < 0.05 corrected for minimum duration criterion), (**d**) GMD statistical differences (p < 0.05 corrected for minimum duration criterion), (**e**) cluster permutation results showing the count of electrodes in the significant positive cluster (p < 0.05) and (**f**) exemplar topography differences (Synch - Baseline and Synch - Asynch) for the peaks of significant electrode count with significant electrodes highlighted in red.

Average omission responses in the Synchronous and Asynchronous conditions were derived from continuous EEG recordings by averaging the peristimulus epochs locked to the first R peak of the electrocardiography (ECG) signal during the time period when sounds were omitted resulting in the so called heartbeat evoked potentials (HEPs, Fig. 1). As a control, we extracted HEPs from the Baseline condition time-locked to a random selection of R peaks. Electrical neuroimaging analysis of HEPs included analysis of Global Field Power (GFP), an index of response strength, and of Global Map Dissimilarity (GMD), an index of difference between conditions in terms of spatial configuration of underlying neural generators^39,40^. Repeated measures ANOVA of HEPs across the three conditions revealed a GFP main effect at 237–270 ms following R peak (p < 0.05) and a GMD main effect at 156–190 ms following R peak (p < 0.05). To identify the origin of the statistical differences in the main analysis, we conducted post-hoc pairwise comparison. Results for the Synchronous vs. Baseline comparison showed a GFP difference at 234–268 ms following R peak (p < 0.05, >28 ms minimum duration, Fig. 2c) and a GMD difference at 233–275 ms following R peak (p < 0.05, >37 ms minimum duration; Fig. 2d). Similarly, comparison of Synchronous vs. Asynchronous conditions revealed a GFP difference at 240–271 ms following R peak (p < 0.05, >25 ms minimum duration, Fig. 2c) and a GMD difference at 158–190 ms following R peak (p < 0.05, >33 ms minimum duration; Fig. 2d). However, no significant GFP or GMD differences were found between Asynchronous vs. Baseline conditions (Fig. 2c,d). These post-hoc results based on GFP and GMD were confirmed by a cluster-permutation test showing for Synchronous vs. Baseline at 153–278 ms and for Synchronous vs. Asynchronous at 148–312 ms following R peak a significant positive cluster in posterior-central scalp regions (cluster level p < 0.05, corrected for multiple comparisons in time and space, Fig. 2e,f). No significant cluster permutation differences were found between Asynchronous vs. Baseline conditions. We conducted correlation analyses to assess whether EEG statistical effects were related to differences in cardio-audio temporal parameters between conditions. The analysis showed no significant correlations of Synchronous vs. Asynchronous difference values between GFP and R peak-to-sound onset (RS) variability (R = 0.10, p = 0.71), GFP and sound onset-to-R peak (SR) variability (R = −0.02, p = 0.93), GMD and RS variability (R = −0.47, p = 0.06), GMD and SR variability (R = −0.21, p = 0.44).

### Omission-Control Analysis

**Figure 3:**
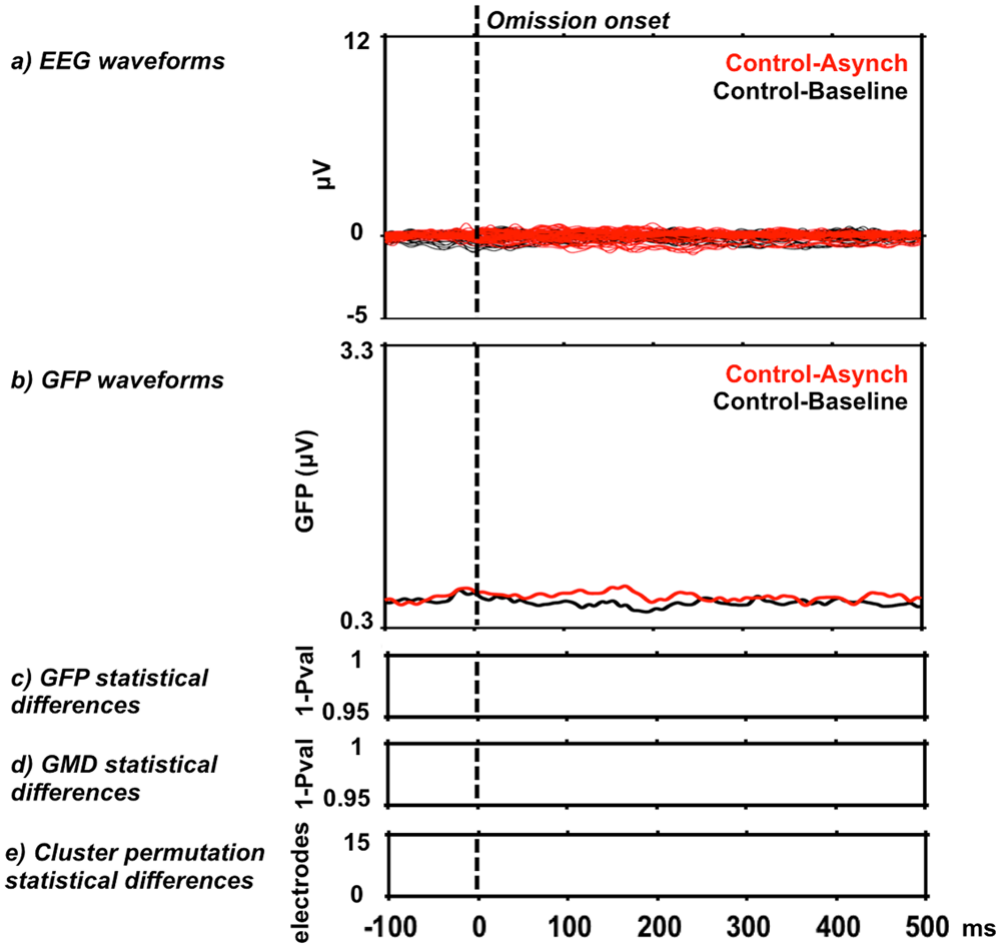
Electrical neuroimaging results for sound-based omission in Asynchronous blocks. (**a**,**b**) Group-average EEG and GFP waveforms of omission trials from Asynchronous blocks (Control-Asynch, in red) with onset at a latency occurring during the omission at the average interstimulus interval. Control-Baseline trials (in black) were randomly selected with onset matched for R peak onset of single trials. (**c**–**e**) Statistical analyses by time-wise GFP, GMD and cluster permutation tests showed no significant differences between sound-based omission in Control-Asynch vs. Control-Baseline condition.

We conducted a control analysis to assess if in the Asynchronous condition an omission response could be observed that depended on the temporal structure of sound sequences, unrelated to heartbeat onset. We thus compared EEG epochs time-locked to omission events from the Asynchronous blocks to Baseline trials matched for the jittered occurrence of heartbeat artifacts. Because the interstimulus intervals of the Asynchronous condition had a normal (Gaussian) distribution and were presented in a random order, temporal prediction of upcoming sounds by participants should have been maximal at the average interstimulus interval. Accordingly, we extracted omission trials from the Asynchronous condition by time-locking event onset to a latency occurring during the omission at the average interstimulus interval (i.e. Control-Asynch condition, no time-locked to R peaks). We compared these data to EEG epochs from the Baseline in which no sounds were presented and no sound prediction was expected. We extracted a random selection of epoch from continuous Baseline recordings (Control-Baseline condition), such that the latencies between epoch onset and closest heartbeat (i.e. R peak) were matched on the single trial level to the trials from the Control-Asynch condition. Comparison of the Control-Asynch to Control-Baseline condition showed no significant differences for GFP analysis, GMD analysis, and cluster-permutation t-test (Fig. 3c–e). A similar analysis comparing a Control-Synch condition to Control-Baseline condition showed no significant results.

### Control Analyses

**Figure 4:**
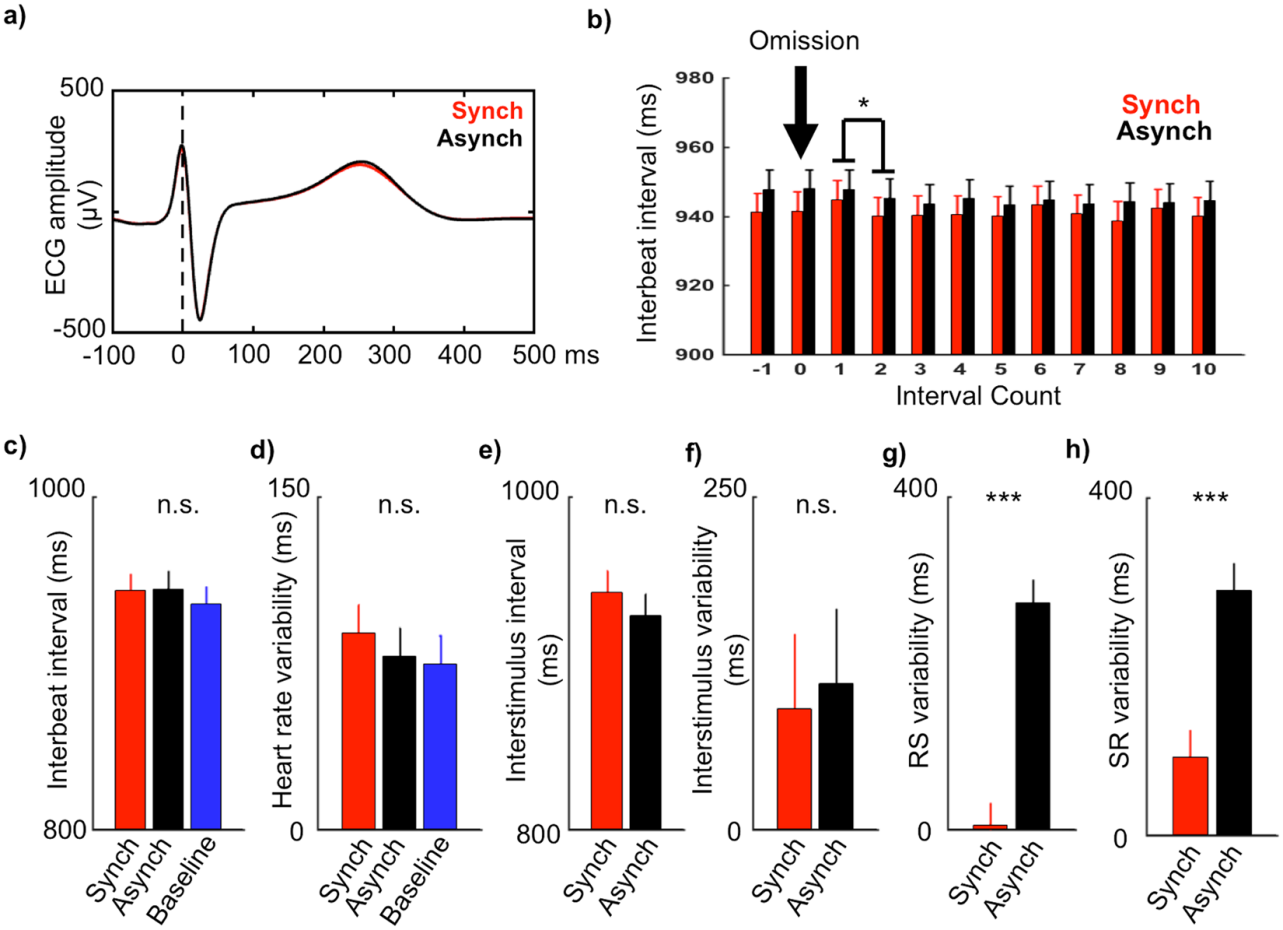
Control analyses results. (**a**) Group-average ECG waveform (N = 16) time-locked to R peaks during sound omission for Synchronous (in red) and Asynchronous (in black) conditions superimposed. (**b**) Interbeat intervals before, during and after omission for Synchronous (in red) and Asynchronous (in black) conditions. (**c**–**h**) Interbeat intervals, heart rate variability, interstimulus intervals, interstimulus variability, RS variability for sound trials, and SR variability for omission trials compared across experimental conditions (*p < 0.05, ***p < 0.001; n.s. = not significant).

Time-wise ECG analysis showed no significant differences between ECG time-locked to R peaks during sound omission in Synchronous versus Asynchronous conditions (p > 0.05; Fig. 4a). Analysis of consecutive interbeat intervals surrounding omission events showed a main effect of Interval (F(11,1) = 2.98, p = 0.001), however no main effect of Condition (F(11,1) = 0.64, p = 0.44) and no Interval x Condition interaction (F(11,1) = 1.01, p = 0.44) were observed. We conducted post-hoc analysis to assess the nature of the Interval main effect. Paired t-tests were calculated on consecutive interbeat intervals averaged over Condition. This analysis showed a duration decrease from Interval “1” to Interval “2” following omission (M_1_ = 946 ms, M_2_ = 942 ms; mean and standard error of pairwise differences: M_DIFF_ = 4 ms, SE_DIFF_ = 1 ms; paired-samples t-test: t(15) = 3.35, p = 0.004) reflecting thus heart rate acceleration immediately after omission in both the Synchronous and the Asynchronous condition (Fig. 4b). Further analyses showed no statistical differences between experimental conditions regarding interbeat interval (Fig. 4c), interbeat variability (Fig. 4d), interstimulus interval (Fig. 4e) and interstimulus variability (Fig. 4f; paired t-test p-values > 0.05). Finally, as expected based on experimental task construction (see Methods), comparison between Synchronous and Asynchronous conditions showed significant differences of RS variability (M_SYNCH_ = 6 ms, M_ASYNCH_ = 273 ms, M_DIFF_ = 320 ms, SE_DIFF_ = 15 ms, paired-samples t-test: t(15) = −21, p < 0.001; Fig. 4g) and SR variability (M_SYNCH_ = 93 ms, M_ASYNCH_ = 290 ms, M_DIFF_ = 237 ms, SE_DIFF_ = 17 ms; paired-samples t-test: t(15) = −13.25, p < 0.001; Fig. 4h).

## Discussion

We investigated whether temporal regularity between interoceptive and exteroceptive bodily signals induces a violation detection response upon stimulus omission. We manipulated the synchrony of sound onset and the participant’s heartbeat and found that sound omission in the Synchronous condition induced both response strength (i.e. GFP), topographical (i.e. GMD), and electrode-level (i.e. cluster permutation t-test) statistical differences of HEPs in the 160–270 ms period following R peak when compared to HEPs from the Baseline and Asynchronous conditions. These differential neural responses between conditions resulted from distinct temporal rules between heartbeat and sound sequences. This manipulation induced variable sound-to-sound intervals following a temporal structure of the participant’s heartbeat rhythm and transient changes due to heart rate variability that were statistically matched across experimental conditions (see section 2.3. Control Analyses and Fig. 4). Thus, the fact that statistical difference were exclusively observed for omission trials time-locked to heartbeats (i.e. Synchronous vs. Baseline and Synchronous vs. Asynchronous) and that no differences were found when comparing omissions on sound-sound temporal regularity (i.e. Control-Asynch vs. Control-Baseline and Control-Synch vs. Control-Baseline) suggests that the temporal structure of sound sequences alone cannot explain the observed results. Instead, our results show that pseudo-random sequences can elicit robust violation detection responses if additional temporal regularity, i.e. cardio-audio synchrony in our case, supports the temporal prediction of future stimuli.

Our study focused on omission responses in order to investigate the violation detection response in absence of any bottom-up contribution of auditory processing and further avoided confounds arising from sequences presented at fixed interstimulus intervals in terms of left over neural responses of previous stimuli or entrainment effects (see^41^ for similar considerations). A previous study by^42^ investigated auditory evoked potentials in response to heartbeat-related sounds versus externally generated sounds and found a marginally significant (p = 0.07) suppression of the N1 component when sound onset was locked to the participant’s heartbeat. However because heartbeat sequences are more regular in comparison to a fully random sequence of externally generated sounds, the N1 suppression could have resulted from differences in the regularity structure of auditory sequences rather than from expectation induced by heartbeat afferent processing. In our study we could exclude this explanation by matching the statistical distribution of the sound-to-sound intervals between Synchronous and Asynchronous conditions. Furthermore, differently from the study by^42^, we controlled for possible contributions of bottom-up components of auditory processing by analyzing omission responses.

Our results can be interpreted in the context of predictive coding^20^. According to this theory the brain constantly monitors the timing of incoming sensory stimuli (bottom-up) and compares these information to a prediction about the onset of future stimuli (top-down). Because here we used variable interstimulus intervals, the brain prediction of upcoming sounds would be temporally imprecise and thus omission of sounds would induce a low prediction error signal, i.e. in line with the absence of effects comparing Control-Asynch to Control-Baseline omission in our study. However, assuming that the brain also monitors the timing of heartbeats, the predictive hierarchy might capitalize on contingencies between heartbeat and sound onset (Synch condition) to generate an accurate prediction about the onset of future sounds. In this case, omission of heartbeat-synchronized sounds would induce a large prediction error, i.e. in line with the statistical differences observed for Synch vs. Baseline and Synch vs. Asynch omission locked to heartbeats. Based on these considerations we propose that our results provide the evidence for predictive coding in time across interoceptive heartbeat and exteroceptive auditory signals.

Notably here we presented a method to avoid possible confounds related to heartbeat physiological artifacts. Indeed, our experiment was designed for comparing neural responses to omissions locked to R peaks. This method has the advantage of studying violation detection without the need of disentangling heartbeat artifacts from neural responses to omitted stimuli. In particular heartbeat evoked potentials have typically been considered as a contaminating contribution to the neural signal of interest, requiring specific control conditions for either removing heartbeat- from auditory evoked activity^42^ or imposing constraints on the time periods of analysis to artifact-free epochs^43^. As it is common to many previous propositions for cleaning the EEG signals from physiological artifacts, such a procedure requires an assumption about the nature of the added contribution of the artifacts to the signal of interest. Physiological heartbeat and also eye blink artifacts are usually dealt with by extracting them from the putative neural signal of interest assuming statistical independency and linear reparability^44^. From a functional perspective the independency between evoked or resting state activity and the neural underpinning of ‘contaminating’ physiological signals remain to be proven. Collectively the process of filtering out heartbeat related physiological activity can sacrifice neural signals and reduces the effect of interest. These arguments make our experimental paradigm particularly suitable for analyzing omission responses without a priori assumptions regarding the interaction between heartbeat and omission responses.

Finally, we would like to point out a few limitations of our study. First, the statistical differences of HEPs between Synchronous and Asynchronous conditions can be confounded by the degree to which the omission response within the auditory sequence temporally coincided with the exact onset of the heartbeat (which -by construction- was not matched between Synchronous and Asynchronous conditions in our study). This question should be addressed by future studies, for instance, by comparing different sound onset latencies with respect to heartbeats. Second, we note that because a randomized sequence of interbeat intervals was used for sound presentation in Asynchronous blocks, the statistical properties over time of the heartbeat sequences were disrupted. Thus, it remains to be investigated by future studies how regularity of heartbeat sequential information influence sound prediction. Third, because in the Synchronous condition omissions were time-locked to heartbeats, we cannot separately evaluate the dependence of the observed statistical difference on neural responses to sound omission, the modulation of neural responses to heartbeats, or the interaction of both processes. Finally, one might ask if our participants were aware of the temporal rules underlying sound presentation during different experimental blocks. All our participants were naïve to the experimental manipulation, as indicated by informal verbal inquiry after the experiment. Indeed, previous studies failed to provide evidence linking spontaneous or task-induced HEP modulations to heartbeat perception^38,42,45,46^.

Taken together, our results suggest that temporal regularities across interceptive and exteroceptive inputs are monitored by the human brain and that these information can potentially drive a violation detection response upon sequence interruption. These results extend earlier proposals regarding interoceptive contributions to time processing^47^ and about the existence of an interoceptive predictive coding mechanism (i.e. see^33,42^ for related proposals). We propose that the brain routinely monitors not only the timing of external or of internal events, but also keeps track of the temporal relation between internal and external stimuli anticipating the timing of future sensory events. Temporal integration across interoceptive and exteroceptive bodily signals might be a central mechanism for optimizing sensory processing, sequence processing, and bodily awareness.

## Materials and Methods

### Ethics Statement

The study protocol was approved by the local ethical committee (La Commission Cantonale d’Ethique de la Recherche sur l’Etre Humain) and the experiment was carried out in accordance with approved guidelines.

### Participants

Twenty volunteers took part in this study. The data of four participants were excluded from analysis because of poor quality of heartbeat recordings leading to a loss of more than 50% of the trials (see below) and the data of the remaining 16 participants were analyzed (6 female, 2 left-handed, average age of 25 years and range from 19 to 34 years). All participants gave their written informed consent and received 60 Swiss Francs for a monetary compensation.

### Stimuli

Sound stimuli were 1000 Hz sinusoidal tones of 100 ms duration and 0 μs inter-aural time difference. A 10-ms linear amplitude envelope was applied at stimulus onset and offset to avoid clicks. Stimuli were 16-bit stereo sounds sampled at 44.1 kHz and were presented binaurally at approximately 70 dB loudness (individually adjusted).

### Experimental Setup

The participant was comfortably seated at a table inside of a sound-attenuated experimental room and was equipped with electrodes for heartbeat, eye movement, and EEG recording (see below). The participant wore in-ear phones (Shure SE 215, Niles, IL) throughout the entire experiment for auditory stimulation and attenuation of external sounds. In-ear phones were used instead of external headphones both to avoid physical contact and thus displacement of EEG cap and electrodes and in order to increase subject comfort. A fixation cross was attached to the wall of the experiment room at eye-level at 1-m distance from the participant.

### Experimental Procedure

Participants were instructed before the experiment to passively listen to sound sequence while fixating the cross in front of them, to avoid body movements and breathe regularly in order to increase the recorded signal quality. They were naïve to the experimental manipulation, as suggested by informal verbal inquiry about the experimental manipulation after the experiment. After the experiment, participants were debriefed about the experimental manipulation (i.e. heartbeat synchronous-asynchronous presentation of sounds) and purpose.

Each participant was presented with three types of conditions presented in separate experimental blocks (i.e. S = Synchronous, A = Asynchronous, and B = Baseline; Fig. 1) in a pseudo-random BSABAS or BASBSA order randomly assigned across participants. This order served to account for temporal effects (i.e. fatigue, habituation) on EEG recordings by reversing the order of subsequent Synchronous-Asynchronous blocks across their first and second repetition (i.e. SA became AS, or vice versa). In addition, each repetition of Synchronous-Asynchronous blocks was preceded by a Baseline (i.e. always as first and fourth experimental block).

The Synchronous condition consisted of sequential presentation of 400 sound stimuli (80% of trials) and 100 omissions (20% of trials) in random order where at least one sound stimulus intervened between two subsequent omissions. Importantly, the temporal onset of each sound stimulus was triggered by an online detection of R peaks from raw electrocardiography (ECG) recordings. A custom MATLAB Simulink script analyzed in real time raw ECG recordings by computing the variance over the preceding 50-ms time window and detected when this value exceed an individually adjusted 10–15 mV^2^ variance threshold which triggered the presentation of a sound stimulus or an omission trial (Fig. 1). This procedure induced in a fixed R peak to sound onset (i.e. RS interval) delay of 41 ms (SD = 12 ms) across participants (see below). Each Synchronous block lasted typically 5–8 minutes depending on the participant’s heart rate. Across all subjects the average interstimulus interval was 936 ms (SD = 176 ms, range: 673–1370 ms). Thus, interstimulus intervals for all subjects were sufficiently large to not cause overlap of subsequent sound stimuli with the main components of the auditory evoked potential.

In the Asynchronous condition, the onset of sound presentation was based on the heartbeat intervals extracted from a preceding Baseline block. Specifically the ECG recorded during the Baseline was analyzed offline to extract interbeat intervals by automatic detection of R peaks and computation of interbeat intervals. Five hundred interbeat intervals were randomly selected and their order was shuffled giving rise to a pseudo-random sequence closely resembling the participant’s heartbeat rhythm. In the Asynchronous blocks sound stimuli (400 trials) and omissions (100 trails) were scheduled according to this predefined pseudo-random sequence. By construction there was virtually no difference between the Synchronous and Asynchronous conditions in terms of average and variance of the interbeat intervals, whereas the RS interval was kept fixed in the Synchronous condition and variable in the Asynchronous condition (mean RS interval: M = 6 ms, SD = 16 ms, range from −29 to 25 ms; RS variability: M = 327 ms, SD = 61 ms, range from 236 to 449 ms). Both these facts were evaluated post hoc in a separate analysis (see section 2.3 Control Analyses). Across all experimental blocks for each participant, a total of 800 sound stimulation and 200 omission trials were presented each for Synchronous and Asynchronous conditions.

### Data Acquisition

Continuous EEG (g.HIamp, g.tec medical engineering, Graz, Austria) was acquired at 1200 Hz from 63 active ring electrodes (g.LADYbird, g.tec medical engineering) arranged according to the international 10–10 system and referenced to the right ear lobe. An additional ECG electrode was attached to the participant’s chest above the heart, a vertical EOG electrode was attached below the right eye, and two bipolar horizontal EOG electrodes were attached to the outer canthi. Impedances of all active electrodes were kept below 50 kΩ and all data were recorded with online 0.1–100 Hz band-pass and 48–52 Hz notch filters.

### Data Preprocessing

Continuous raw data was band-pass filtered (1 Hz low cut-off, 40 Hz high cut-off, ripple: 0.05 dB, attenuation: 80 dB, transition bandwidth: 0.5 Hz) using second-order Butterworth filters. Artifact electrodes were identified using a signal variance criterion (3 z-score Hurst exponent) and interpolated using spherical splines^48^. On average, 8.75 (SD = 2.44, range = 5–13) electrodes (M = 13.89%, SD = 3.87%) were interpolated for each participant. The data was recalculated against the average reference and EEG epochs from −100 to 500 ms relative to event onset were extracted. The epoch duration was determined according to the timing of omission responses in previous EEG studies and by choosing a maximum duration that would not cause overlap with subsequent heartbeats (i.e. assuming a 60–100 beats-per-minute resting rhythm)^13,16,17^. For the different purposes of each experimental analysis (see sections 4.8.1–4.8.3) we used as event onsets either sound onsets, omission onsets, or R peak latencies. R peaks were detected offline using a semiautomatic approach: First, a custom MATLAB script (i.e. “rpeakdetect.m” function; https://ch.mathworks.com/matlabcentral/fileexchange/72-peakdetect-m/content/peakdetect.m) automatically detected the steep positive peaks in continuous ECG data. Second, we performed visual inspection of all data to assure correct detection of R peaks using as visual references the positive P and T waves in which the QRS complex is embedded. No pre-stimulus baseline correction was applied for each participant and each experimental condition. Epochs containing physiological artifacts (e.g. eye movement, excessive muscle activity) were identified using a ±100 μV threshold applied to the EOG and EEG channels and by visual inspection and were excluded from further analysis. For subsequent analysis for each participant we matched the number of accepted trials between experimental conditions by random selection. Across subjects a total of 180 ± 18 omission trials (i.e. Mean ± SD; out of 200 epochs in total) were accepted for the Synch, Asynch, Baseline, Control-Asynch, and Control-Baseline conditions.

### Electrical Neuroimaging Analysis

Reference-independent analyses of topographical changes (i.e. GMD,^49^) and strength modulations (i.e. GFP^50^) of the global electrical field across conditions were conducted using the MATLAB (R2015b, The MathWorks, Natick, MA) toolboxes EEGLAB (release 13.5.4b, http://sccn.ucsd.edu/wiki/Main_Page), Ragu (http://www.thomaskoenig.ch/Ragu_pkg.exe), and FieldTrip^51^ as well as custom scripts. GMD^49^ is calculated as the root mean square of the differences between two strength-normalized vectors (i.e. instantaneous voltage potentials across the electrode montage). The GMD values between experimental conditions (i.e. between factor levels of the Condition factor) were then compared at each time point with an empirical distribution derived from a bootstrapping procedure (5000 permutations per data point) based on randomly reassigning each participant’s data to either one of the two experimental conditions. GFP is calculated as the square root of the mean of the squared values recorded at each electrode (versus average reference) and represents the spatial standard deviation of the potentials at all electrodes and at each time point^39^. This measure indicates the global strength of the response, regardless of its topographic distribution. Changes in GFP were statistically analyzed by one-factorial repeated-measures ANOVAs (i.e. number of factors levels depended on analysis type: see below) using an a priori alpha threshold of p < 0.05. We corrected for temporal autocorrelation by using the ‘duration count estimate’ option in the Ragu toolbox^49^. The latter evaluates whether an observed duration of a period of significant differences in randomization statistics would have been observed by chance and is computed based on the results of randomization runs of each analysis (see^49^ for further detail). Estimations of duration counts across analyses in our study ranged between 26–35 ms minimum duration for the −100 to 500 ms interval relative to event onset.

We complemented the electrical neuroimaging analysis with a cluster-based permutation t-test^52^ for a confirmation of the results obtained with the methods presented above at electrodes level. This analysis was applied for evaluating the EEG differences between experimental conditions. Individual data samples showing significant t-values (p < 0.05, two-tailed) were clustered based on temporal and spatial proximity and each cluster was assigned to cluster-level statistics corresponding to the sum of the t-values of the samples belonging to that cluster. The type-I error rate was controlled by evaluating the maximum cluster-level statistics by randomly shuffling condition labels 1000 times to estimate the distribution of maximal cluster-level statistics obtained by chance and applying a two-tailed Monte-Carlo p-value. This procedure was applied at the sensor level in the time window from −100 to 500 ms relative event onset.

Post-hoc correlation analyses assessed whether the statistical differences in EEG between Synchronous and Asynchronous condition were related to cardio-audio temporal parameters. For each participant we extracted the average GFP difference and the average GMD between Synchronous and Asynchronous conditions across the temporal intervals where statistical differences were observed. As indices of cardio-audio synchronization we computed the Synch-Asynch difference for both the SR variability and RS variability. These data were subjected to separate linear correlation analyses between GFP and RS, GFP and SR, GMD and RS, GMD and SR (alpha threshold of 0.05, uncorrected).

#### Omission Response Analysis

The main analysis of this study tested whether temporal synchronization of sounds and heartbeats in the Synchronous (Synch) condition induced temporal expectation of sounds and thus a violation detection response upon omission. Accordingly, we extracted from the Synchronous and Asynchronous conditions the heartbeat-evoked potentials (HEPs) time-locked to the first R peak (i.e. based on ECG recording) during the omission (see Fig. 1 for illustration). In addition, we extracted HEPs from a random selection of heartbeats from resting state Baseline recordings, where half of the trials were extracted from the first and the other half of the trials from the second Baseline block. We tested the hypothesis that violation detection upon omission of heartbeat-synchronized sounds should emerge as an evoked neural response different from HEPs during omission in asynchronous cardio-audio sequences and different from resting EEG. GFP and GMD across the three conditions were analyzed with time-running repeated-measures ANOVAs with the within-subjects factor Condition (levels: Synchronous, Asynchronous, Baseline). Next, we identified the conditions underlying the effects from the main analysis by pairwise post-hoc comparisons between Synchronous vs. Baseline, Synchronous vs. Asynchronous, and Asynchronous vs. Baseline conditions.

#### Omission-Control Analysis

By construction, in our experimental design the sound-to-R peak (SR) interval for omission trials was more variable in the Asynchronous than in the Synchronous condition (see also 3.4 Control analyses and Fig. 4h). This difference might have induced statistical differences between Synch and Asynch conditions in the Omission Response analysis independent of the effect of interest, i.e. sound prediction based on cardio-audio temporal synchronization in the Synchronous condition. Indeed we cannot exclude that the pseudo regularity of the heartbeat (and therefore of the auditory sequence) would be sufficient to induce a violation detection response in the Asynch and in the Synch conditions and that the effect observed in the Omission Response analysis resulted from unmatched SR intervals.

Thus, in the Omission-Control analysis we tested whether a violation detection response was evoked in the Asynchronous condition based on sound-sound temporal regularity independent of heartbeats. We extracted from the Asynchronous condition the EEG evoked potentials time-locked to the average interstimulus interval during omission interval. We note that this time point corresponded to the maximum probability of sound occurrence based on interstimulus interval distribution function (Control-Asynch condition). For comparison we extracted EEG evoked potentials from the Baseline condition (i.e. no sounds were presented) that were matched for the presence of heartbeat onset at the single trial level (Control-Baseline condition). We hypothesized that if temporal prediction based on sounds (and independent of heartbeats) was present in the Asynchronous condition, EEG statistical differences shall be observed between Control-Asynch and Control-Baseline condition (Table 1). Alternatively, no statistical differences between these conditions would indicate no sound-based temporal prediction in the Asynchronous condition, We interpret this result in the light of the large interstimulus variability (see 3.4 Control analyses and Fig. 4f). Statistical analysis of these data was carried out using a one-factorial repeated-measures ANOVA with the factor Condition (levels: Control-Asynch, Control-Baseline).

**Table 1:** Hypotheses related to the three main analysis of this study.

Analysis	Hypothesis and description	
Omission Response Analysis	**H1**	**Omission response time-locked to heartbeats during cardio-audio synchronous but not asynchronous stimulation**.
	H0	No omission responses time-locked to heartbeats.
Omission-Control Analysis	**H1**	**Omission response time-locked to the average interstimulus interval (maximum stimulus probability) during cardio-audio asynchronous stimulation**
	H0	No omission response time-locked to the average interstimulus interval.
Control Analyses	**H1**	**Presence of heartbeat physiological or temporal differences, or sound-sound temporal differences between cardio-audio Synchronous and Asynchronous conditions (Possible confounds for the interpretation of EEG effects as of neural origin)**.
	H0	No heartbeat physiological, temporal, or sound-sound temporal differences between cardio-audio Synchronous and Asynchronous conditions

### Control Analyses

Finally, we performed control analyses to assess if changes in heartbeats itself contributed electrical neuroimaging results^43,53^. Event-related averages of the ECG for omission trials of the Synchronous, Asynchronous, and Baseline condition were calculated and statistically analyzed with a time-running one-factorial repeated-measures ANOVA (i.e. within-subjects factor Condition; levels: Synchronous, Asynchronous, Baseline) using an alpha threshold of p < 0.05 and a >32-contiguous-time-points (>26 ms) temporal criterion to correct for temporal autocorrelation. Next, we tested whether sound omission induced changes of the heartbeat rhythm. For the Synchronous and Asynchronous condition we extracted twelve consecutive interbeat intervals around each omission event, ranging from one heartbeat before omission (“−1” label) to ten heartbeats following omission (“10” label; i.e. “0” refers to the interbeat interval immediately preceding the omission). We calculated for each interval and experimental condition the individual median interbeat interval and subjected these data to across-subjects statistical analyses using a 12 (Interval: “−1” to “10”) × 2 (Condition: Synchronous, Asynchronous) repeated measures ANOVA.

We also analyzed if there were overall differences in interbeat intervals and interstimulus intervals across experimental conditions that might have confounded the present results. For this, we extracted interbeat intervals and interstimulus intervals (for sound trials preceded by sound trials) for the Synchronous, Asynchronous, and Baseline conditions. Statistical comparisons were made using paired t-tests (alpha < 0.05). Finally, we extracted RS intervals for sound trials, an index of cardio-audio synchronization, and SR intervals for the first heartbeat in the omission period, in order to quantify heartbeat onset variability during omission. We compared the distributions of RS intervals and SR intervals to investigate whether our method induced the desired temporal regularity-irregularity between heartbeat and sound onset. For this, we computed for each participant and each condition the standard deviation across RS intervals and statistically analyzed these data using paired t-tests (alpha < 0.05).

### Data Availability

The datasets analyzed during the current study are available from the corresponding author on reasonable request.
